# Preparation and Application of Amino-Terminated Hyperbranched Magnetic Composites in High-Turbidity Water Treatment

**DOI:** 10.3390/molecules28196787

**Published:** 2023-09-24

**Authors:** Yuan Zhao, Qianlong Fan, Yinhua Liu, Shuwen Wang, Xudong Guo, Liujia Guo, Mengcheng Zhu, Xuan Wang

**Affiliations:** 1 College of Chemistry and Chemical Engineering, Henan University of Science and Technology, Luoyang 471000, China; 2 College of Basic Medicine and Forensic Medicine, Henan University of Science and Technology, Luoyang 471000, China

**Keywords:** hyperbranched polymer, Fe_3_O_4_, coating, magnetic separation, water treatment

## Abstract

In order to separate the colloidal in high-turbidity water, a kind of magnetic composite (Fe_3_O_4_/HBPN) was prepared via the functional assembly of Fe_3_O_4_ and an amino-terminal hyperbranched polymer (HBPN). The physical and chemical characteristics of Fe_3_O_4_@HBPN were investigated by different means. The Fourier Transform infrared spectroscopy (FTIR) spectra showed that the characteristic absorption peaks positioned at 1110 cm^−1^, 1468 cm^−1^, 1570 cm^−1^ and 1641 cm^−1^ were ascribed to C–N, H–N–C, N–H and C=O bonds, respectively. The shape and size of Fe_3_O_4_/HBPN showed a different and uneven distribution; the particles clumped together and were coated with an oil-like film. Energy-dispersive spectroscopy (EDS) displayed that the main elements of Fe_3_O_4_/HBPN were C, N, O, and Fe. The superparamagnetic properties and good magnetic response were revealed by vibrating sample magnetometer (VSM) analysis. The characteristic diffraction peaks of Fe_3_O_4_/HBPN were observed at 2θ = 30.01 (220), 35.70 (311), 43.01 (400), 56.82 (511), and 62.32 (440), which indicated that the intrinsic phase of magnetite remained. The zeta potential measurement indicated that the surface charge of Fe_3_O_4_/HBPN was positive in the pH range 4–10. The mass loss of Fe_3_O_4_/HBPN in thermogravimetric analysis (TGA) proved thermal decomposition. The –C–NH_2_ or –C–NH perssad of HBPN were linked and loaded with Fe_3_O_4_ particles by the N–O bonds. When the Fe_3_O_4_/HBPN dosage was 2.5 mg/L, pH = 4–5, the kaolin concentration of 1.0 g/L and the magnetic field of 3800 G were the preferred reaction conditions. In addition, a removal efficiency of at least 86% was reached for the actual water treatment. Fe_3_O_4_/HBPN was recycled after the first application and reused five times. The recycling efficiency and removal efficiency both showed no significant difference five times (*p* > 0.05), and the values were between 84.8% and 86.9%.

## 1. Introduction

The water environment is of vital importance to the sustainable development of mankind [1]. In light of the rapid industrial development, urbanization and growing utilization of chemical materials, as well as the increased concentration of raw turbidity and suspended sediment in water treatment caused by extreme weather (heavy precipitation and floods), water treatment is facing unprecedented challenges, such as black odor, eutrophication, etc. [2,3,4]. Therefore, it is of great significance to study the separation of suspended particles in high-turbidity water. Kaolin is a kind of typical mineral, and abundant colloidal particles exist in its suspension. The colloidal particles increase the turbidity in solution and are difficult to settle under gravity alone due to the electrostatic interactions.

The major methodologies currently used for separating suspended particles include filtration, centrifugation, flotation, electrophoresis, flocculation, etc. [5,6,7]. Flocculation is often applied as a common method, which can be defined as the process by which a solute particle in a solution forms aggregates named flocs. The flocculation process can occur in the mechanisms acting alone or in combination with charge neutralization, electrostatic patch, bridging and sweeping flocculation [8,9,10]. Magnetic flocculation is a branch of the flocculation technology that removes the pollutants by reaction with magnetic flocculant. The strategies are based on the replacement of traditional flocculant in water treatment by magnetic flocculant. Due to the addition of the magnetic materials, the flocs characterized by a high magnetic susceptibility are formed and separated with the external magnetic field. Moreover, the settling velocity of magnetic floc is much faster under the influence of magnetic force than that in the situation of gravity [11].

In the process of magnetic flocculation separation, the selection of magnetic flocculant plays a key role in improving the separation effect. Typically, it is obtained by the functional assembly of magnetic materials with conventional flocculants, which combines both characteristics of magnetic separation and flocculation simultaneously [12]. Fe_3_O_4_ is a common magnetic material which can be used for the functional assembly of flocculants. But single Fe_3_O_4_ nanoparticles are usually insoluble in water, and the flocculation effect could be affected due to agglomeration and precipitation [13,14]. After functional assembly, the resulting magnetic composites not only improve the surface charge and polymerization properties of Fe_3_O_4_ but also improve the electrophoretic mobility and isoelectric point [15]. Polymers including inorganic polymers, organic polymers, and biopolymers are often used in the assembly of magnetic materials [16,17,18]. Most of these polymers are linear or chained with one or more –N group functional groups (e.g., –NH_2_, –CONH_2_, –N^+^) in the main or branch chains. These functional groups can be grafted onto the surface of Fe_3_O_4_ by electrostatic force and Van Der Waals force to form active sites and improve magnetic separation efficiency [19]. Among the polymers, hyperbranched polymers (HBP) show great potential for the functional assembly with Fe_3_O_4_ due to the highly branched and unique three-dimensional configuration [20,21,22].

In the three-dimensional quasi-spherical structure of HBP, a large number of internal cavities and active –N groups are filled. In functional assembly with Fe_3_O_4_, supramolecular assemblers with multiple force forms (Van Der Waals forces, hydrogen bonds) are constructed based on the non-covalent interactions of the three-dimensional super-branching structure [23,24,25]. When reacted with the target pollutant, the magnetic composites showed excellent removal properties by forming electrostatic interactions and hydrogen bond interactions with the analytes [26,27,28]. The magnetic composites obtained by an embedded assembly of HBP and Fe_3_O_4_ improve the assembly and regeneration stability [29]. In addition, the risk of the active site being replaced or complex reaction during water treatment is overcome, and the adsorption performance of the material is maintained [30].

In the research, amino-terminated hyperbranched magnetic composites were preparation by an embedded assembly of three-dimensional HBPN and Fe_3_O_4_. The physical and chemical characteristics were analyzed with the methods of FTIR, scanning electron microscopy (SEM), EDS, VSM, XRD, XPS, zeta potential and TGA. Meanwhile, the separation properties of the magnetic nanocomposite were evaluated by applying to the removal and separation of suspended particles in high turbidity water. Also, the recycling and reusing effects were explored.

## 2. Results and Discussion

### 2.1. Characterization of Fe_3_O_4_/HBPN

#### 2.1.1. FTIR

**Figure 1:**
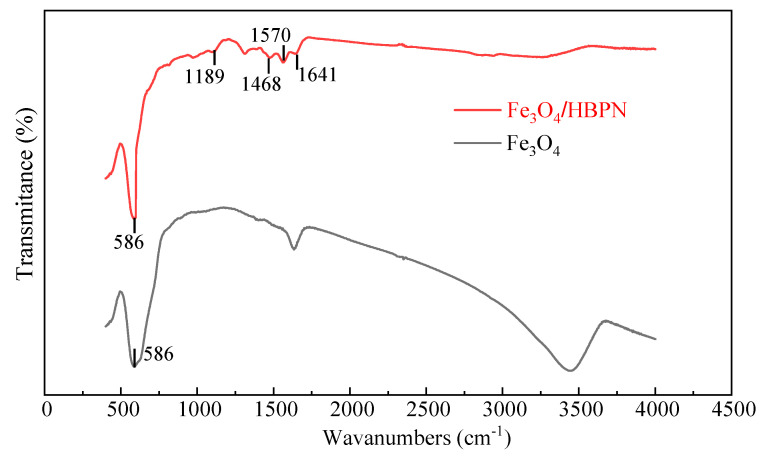
FTIR spectra of Fe_3_O_4_ and Fe_3_O_4_/HBPN.

The FTIR spectra of Fe_3_O_4_ and Fe_3_O_4_/HBPN are shown in Figure 1. The absorption peak at 586 cm^−1^ was attributed to the vibration peak of Fe–O [31,32,33]. For the sample of Fe_3_O_4_/HBPN, the peak at 1468 cm^−1^ was caused by the bending vibration of the H–N–C bond connected to the amino group [34,35]. In addition, the characteristic absorption peaks corresponding to the C=O and N–H stretching vibrations were observed at 1641 cm^−1^ and 1570 cm^−1^ due to the created amido bond [36]. A small peak at 1189 cm^−1^ was ascribed to C–N stretching vibration [37,38]. The typical functional bonds of the amino functional group were detected on the spectra of Fe_3_O_4_/HBPN in the corresponding position, which indicated that the amino functional group was coated on the surface of Fe_3_O_4_/HBPN successfully.

#### 2.1.2. XRD

**Figure 2:**
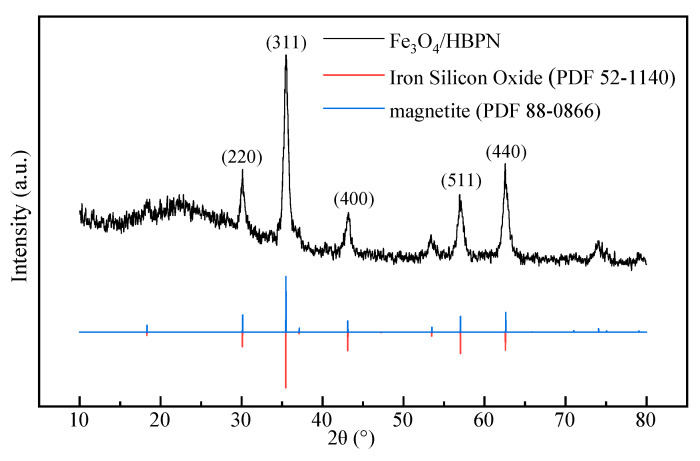
X-ray diffraction pattern of Fe_3_O_4_/HBPN.

In order to analyze the crystalline structure of Fe_3_O_4_/HBPN composites, analysis of the XRD spectra of Fe_3_O_4_/HBPN was carried out. In the XRD patterns shown in Figure 2, Fe_3_O_4_/HBPN showed characteristic diffraction at 2θ = 30.01°, 35.70°, 43.01°, 56.82°, and 62.32°, corresponding to crystal planes (220), (311), (400), (511), and (440), respectively. The position and relative intensity of the diffraction peaks suitably matched those of the JCPDS card (88-0866) for magnetite [39,40]. This meant that Fe_3_O_4_/HBPN still remained an intrinsic phase of magnetite. In addition, the diffraction peaks also matched the JCPDS card (52-1140) for iron silicon oxide [41]. The silicon in Fe_3_O_4_/HBPN came from the APTMs, which was one of the most important ingredients for preparation. This result also corroborated the results of EDS.

#### 2.1.3. XPS

**Figure 3:**
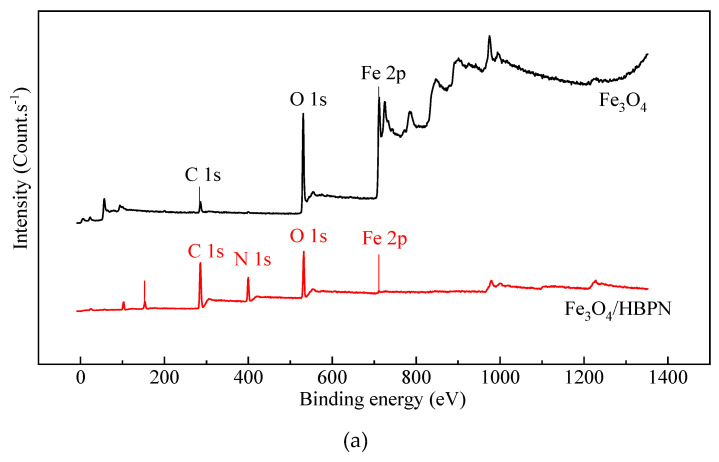

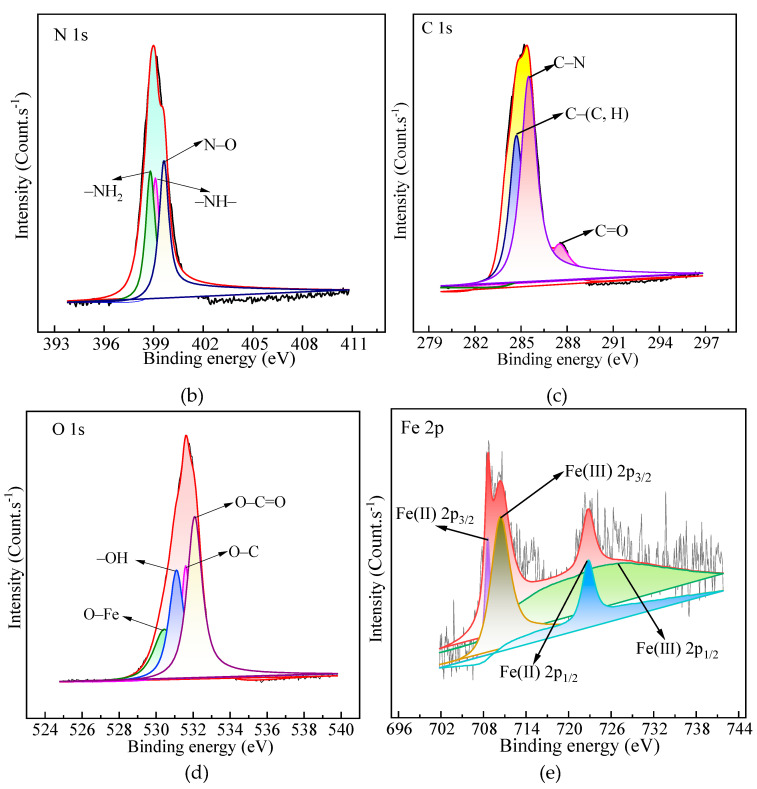
X-ray photoelectron spectra (XPS) of Fe_3_O_4_ and Fe_3_O_4_/HBPN in the survey scan (**a**) and high-resolution XPS spectra of Fe 2p (**b**), C 1s (**c**), N 1s (**d**) and O 1s (**e**) peaks on the surfaces of Fe_3_O_4_/HBPN.

In order to investigate the elemental composition, the chemical oxidation states of surface and near-surface species, the XPS of Fe_3_O_4_/HBPN in the survey and high-resolution narrow scan are presented, respectively (Figure 3). From the survey scan in Figure 3a, a new peak owing to N 1s could be observed on the Fe_3_O_4_/HBPN spectra, which is assigned to the constituent elements of HBPN coated on Fe_3_O_4_ surface.

The N 1s high-resolution scan of Fe_3_O_4_/HBPN can be deconvoluted into three individual peaks at binding energies of 398.6 eV, 399.1 eV and 400.2 eV (Figure 3b), which were assigned to the N atoms in the C–NH_2_, C–NH, and N–O groups [42,43,44], respectively. In the C 1s spectrum (Figure 3c), the C 1s peak in Fe_3_O_4_/HBPN was decomposed into three subpeaks at 284.9 eV, 285.6 eV and 287.9 eV, respectively. The peak at 284.9 eV belonged to the C–(C, H) from hydrocarbon-like compounds [45]. The second peak 285.6 was attributed to the –C or C–N or O–C–O bonds in amide polymers [46]. The third peak 287.6 eV corresponded to the C=O in groups from carboxylate [47]. As illustrated in Figure 3d, the peaks at 529.9 eV and 531.2 eV were, respectively, related to the chemical bonds between oxygen atoms and the Fe and –OH groups on the magnetic composite surface [48]. Also, the peaks related to 532.0 eV and 533.1 eV contributed to the lattice oxygen in Fe_3_O_4_ and bidentate species (O–C=O), respectively [49,50].

As for Fe 2p peaks (Figure 3e), the two main peaks at 710.8 eV and 724.6 eV were attributed to Fe 2p_3/2_ and Fe 2p_1/2_ with peak areas of 59.6% and 40.4%, respectively. Also, the two peaks Fe 2p_3/2_ and Fe 2p_1/2_ were deconvoluted into Fe^3+^ and Fe^2+^, and two doublets were split. Specifically, the peaks centered at 709.9 eV and 723.7 eV were, respectively, attributed to Fe^2+^ 2p_3/2_ (15.1%) and Fe^2+^ 2p_1/2_ (16.2%) split orbitals. Meanwhile, the deconvoluted peaks at 712.6 eV and 727.0 eV were attributed to Fe^3+^ 2p_3/2_ (30.4%) and Fe^3+^ 2p_1/2_ (38.7%) split orbitals, correspondingly [51,52,53]. The Fe^2+^/Fe^3+^ ion ratio for Fe 2p_3/2_ was found to be 0.49, which was close to 0.50, which was obtained from the atomic ratio contained in the naked Fe_3_O_4_ particles. The above-mentioned results confirmed the presence of amine groups from HBPN in the synthesized Fe_3_O_4_/HBPN composites, and the coating mechanism of Fe_3_O_4_/HBPN composites was mainly due to the –C–NH_2_ or –C–NH linkages of HBPN polymers, which bonded to the Fe_3_O_4_ particles via the N–O bonds in the convolving process between loading sites of Fe_3_O_4_ and HBPN polymers.

#### 2.1.4. SEM and EDS

**Figure 4:**
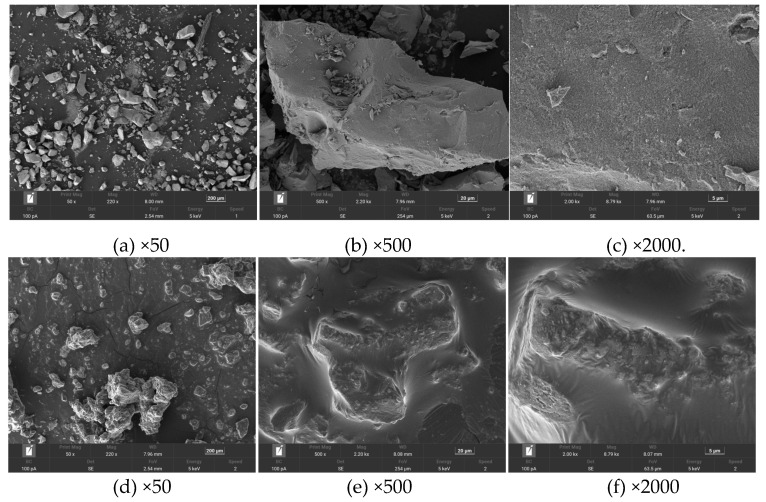
SEM images of Fe_3_O_4_ (**a**–**c**) and Fe_3_O_4_/HBPN (**d**–**f**).

The surface morphology of Fe_3_O_4_ and Fe_3_O_4_/HBPN was determined by FE-SEM (Figure 4a–f). The Fe_3_O_4_ particles presented an irregular granular morphology with different sizes, a block-like structure, a smooth surface and wrinkled edges. The irregular granular morphology may be owing to the co-precipitation method of Fe_3_O_4_ preparation [54]. In contrast, the Fe_3_O_4_/HBPN composites clumped together and were loaded with an oil-like film after HBPN coating (Figure 4e,f). The high magnification image of Fe_3_O_4_/HBPN showed that the membranous-like structure was enveloped and wrapped around the Fe_3_O_4_ particles. These membranous-like structures on the Fe_3_O_4_/HBPN surface may be formed due to the high viscosity and high polymerization characteristics [55,56].

The surface elements of Fe_3_O_4_ and Fe_3_O_4_/HBPN are shown by the EDS spectrum analysis. As shown in Table 1, the main elements of the Fe_3_O_4_ sample are Fe and O. The wt% ratio of Fe and O was 2.87, which proved the successfully prepared of Fe_3_O_4_. For the Fe_3_O_4_/HBPN, the main elements were C, N, O, Si and Fe. Relative to Fe_3_O_4_, the appearance of C and N elements illustrated that the results of elemental analysis were consistent with the composition characteristics of the material, which further verified the successful synthesis of the material.

#### 2.1.5. TGA

**Figure 5:**
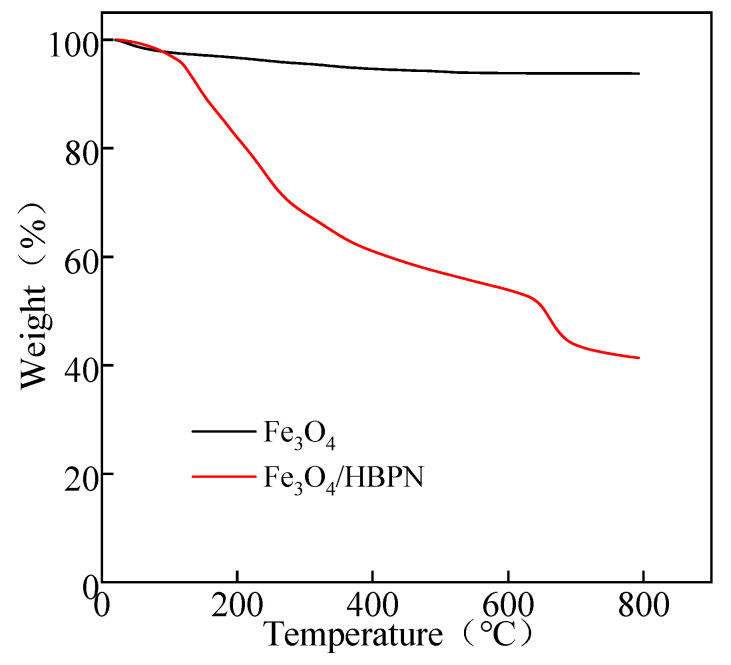
Thermogravimetric curves of Fe_3_O_4_ and Fe_3_O_4_/HBPN.

TGA is a process used to heat the analyzed samples and decompose them by breaking their chemical bonds, which is completed in order to assess the effect of thermal weightlessness and thermal stability [49,57,58]. Figure 5 shows the weight loss curves of Fe_3_O_4_ and Fe_3_O_4_/HBPN against temperature changes, respectively. The weight loss of Fe_3_O_4_ at 20–500°C was about 6%, revealing a high content of Fe_3_O_4_ with little impurity and humidity on its surface. In the temperature range of 500–800 °C, 0.9% mass loss appeared due to the thermal decomposition of the magnetite residue [59]. The results demonstrated that the Fe_3_O_4_ particles exhibited excellent thermal stability.

The weight loss of Fe_3_O_4_/HBPN was analyzed through three stages at different temperature ranges. In the range of 20–110 °C, the weight loss was 3%, which was caused by the thermal decomposition of residual organic solvents during the assembly process. There was a rapid weight loss (45%) in the range of 110–640 °C, which was due to the degradation of super-branched amino groups and alkyl [60]. Following, the weight loss was 10% from 500 to 800 °C, which was due to the chain-breaking thermal decomposition of HBPN, which was gradually carbonized. Overall, the mass loss curve of Fe_3_O_4_/HBPN exhibited an approximately linear trend, indicating that a constant weight loss occurred over the 20–800 °C ranges. Because the thermal stability of the composites mainly depends on the mass of the organic chemical groups, which was coated onto the surface of the target objects [61]; thus, the super-branched amino groups of –C–NH_2_ and –C–NH will induce the degradation with increasing temperature.

#### 2.1.6. VSM

In order to study the magnetic properties of Fe_3_O_4_ and Fe_3_O_4_/HBPN particles, the magnetic hysteresis loop was investigated; thus, the parameters of saturation magnetization (*Ms*), remanent magnetization (*Mr*), and coercive force (*Hc*) could also be extracted. *Ms* means the maximal magnetization value of magnetic particles, which magnetized as an external magnetic field was applied. Following, as the external magnetic field was removed, the magnetic particles still retained their remanent magnetization, which was named *Mr*. In order to remove the remanent magnetization, an external field with an opposite direction of *Mr* will be applied, and the strength of the opposite magnetic field is named *Hc* [62,63,64]. Also, the value of *Hc* exhibited the difficulty level of the magnetic particles to be magnetized.

**Figure 6:**
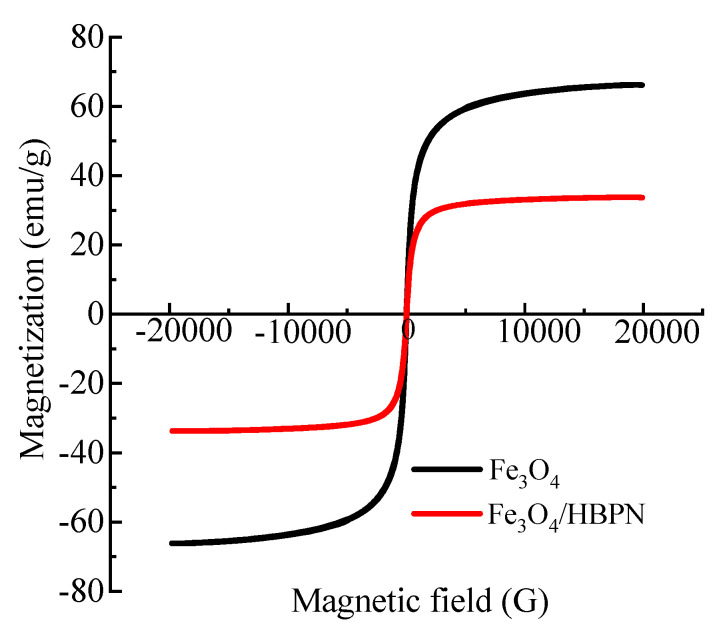
Magnetization hysteresis loops of Fe_3_O_4_ and Fe_3_O_4_/HBPN.

In order to evaluate the magnetic properties, the Fe_3_O_4_ and Fe_3_O_4_/HBPN samples were tested by VSM. As shown in Figure 6, the saturation magnetization (*Ms*) value of the Fe_3_O_4_ was 66.1 emu/g, while the *Ms* value of Fe_3_O_4_/HBPN was attenuated to 33.7 emu/g. This was possibly because of the presence of amino-terminated hyperbranched polymers coated on Fe_3_O_4_, and the polymers were non-magnetic. However, the *Ms* value of the Fe_3_O_4_/HBPN indicated that it was high enough to meet the requirement of magnetic separation by an external magnetic field. Moreover, the curves passed the origin of coordinates, which indicated that the coercivity (*Hc*) and residual magnetization (*Mr*) were close to zero, and there was almost no residual magnetic generation. It showed that Fe_3_O_4_/HBPN had superparamagnetic properties and a good magnetic response, so it was convenient for separation, recycle and reuse by recovering through an external magnetic field.

#### 2.1.7. Zeta Potential

**Figure 7:**
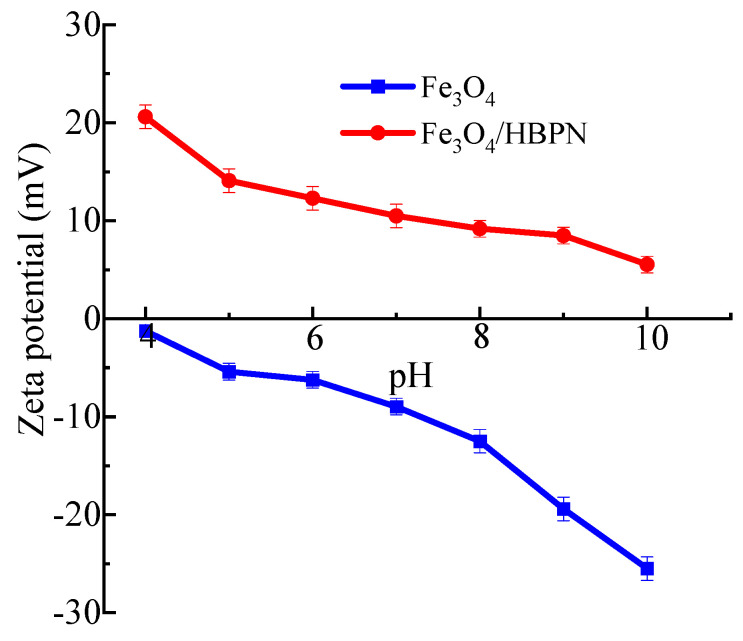
Zeta potential of Fe_3_O_4_ and Fe_3_O_4_/HBPN.

The zeta potential mainly investigates the occurrence of electric potential between the colloidal particles and bulk liquid, which causes the suspension of colloids [65]. As shown in Figure 7, the characteristic surface charge of Fe_3_O_4_ and Fe_3_O_4_/HBPN was investigated by zeta potential measurement. The results showed that Fe_3_O_4_ exhibited a negative charge in the pH range 4–10. On the contrary, Fe_3_O_4_/HBPN showed a positive charge in the same pH range. This indicated that the zeta potential changed from negative to positive when the amino-terminated hyperbranched polymer assembled on Fe_3_O_4_. When the naked Fe_3_O_4_ particles were dispersed in distilled water, the Fe_3_O_4_ surface captured more H^+^ ions than OH^−^. The hydrogen ions adsorbed on the Fe_3_O_4_ surface, forming hydroxyl groups, which resulting in the naked Fe_3_O_4_ being negatively charged [66,67,68]. In addition, the H^+^ from hydroxyl groups can react with amine groups such as –NH, –NH_2_, –N^+^ and –N(CH_3_) [69,70]. In the Fe_3_O_4_/HBPN, the dendritic structure with –NH_2_ displaced H^+^ ions on the Fe_3_O_4_ surface, and the ionic exchange resulted in a positive charge on the Fe_3_O_4_ surface, changing the zeta potential of Fe_3_O_4_/HBPN to positive. Thus, the zeta potential of Fe_3_O_4_/HBPN was positive.

### 2.2. Application Performance of Fe_3_O_4_/HBPN

#### 2.2.1. Effects of Dosage

**Figure 8:**
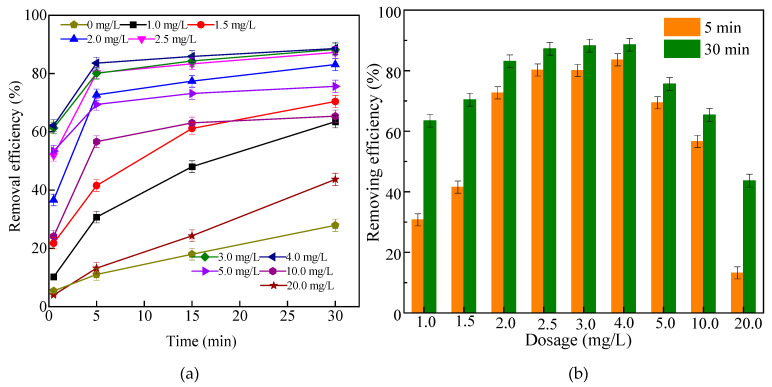
The effect of different dosages on kaolin suspension treatment at diverse times (**a**) and fixed times (**b**). (Ph = 5.6, kaolin suspension concentration was 1.0 g/L, magnetic field intensity was 3800 G).

The kaolin-simulated high-turbidity wastewater was used to investigate the performance of Fe_3_O_4_/HBPN, and different influence factors including dosing concentration, pH, kaolin suspension concentration, magnetic field intensity were explored. As observed in Figure 8a, although the removal effect of all the dosages was increased before 5 min, there was already a noticeable difference. When the Fe_3_O_4_/HBPN concentration was 2.5 mg/L, the removal efficiency of kaolin was 80% in 5 min, and it reached 87% in 30 min (Figure 8b). In the first echelon, the removal effect was similar to 3.0 mg/L and 4.0 mg/L. However, with the increase in Fe_3_O_4_/HBPN concentration, the removal effect was worse, and the kaolin removal efficiency was 44% in 30 min.

According to the zeta potential, the surface of Fe_3_O_4_/HBPN was positively charged, which neutralized and reacted with the negative charges on the surface of kaolin. The large kaolin magnetic flocs were formed and then trapped smaller flocs through the sweeping effect. At last, all magnetic flocs moved and settled along the magnetic field. In addition, the amino groups of Fe_3_O_4_/HBPN provided a large amount of adsorption sites to enhance the removal effect by bridging action, simultaneously. Unfortunately, when the Fe_3_O_4_/HBPN concentration was too high, a large electrostatic repulsion among the initially flocs was formed, resulting in the instability of the flocs and difficulty of generating and aggregating [71].

#### 2.2.2. Effects of pH

**Figure 9:**
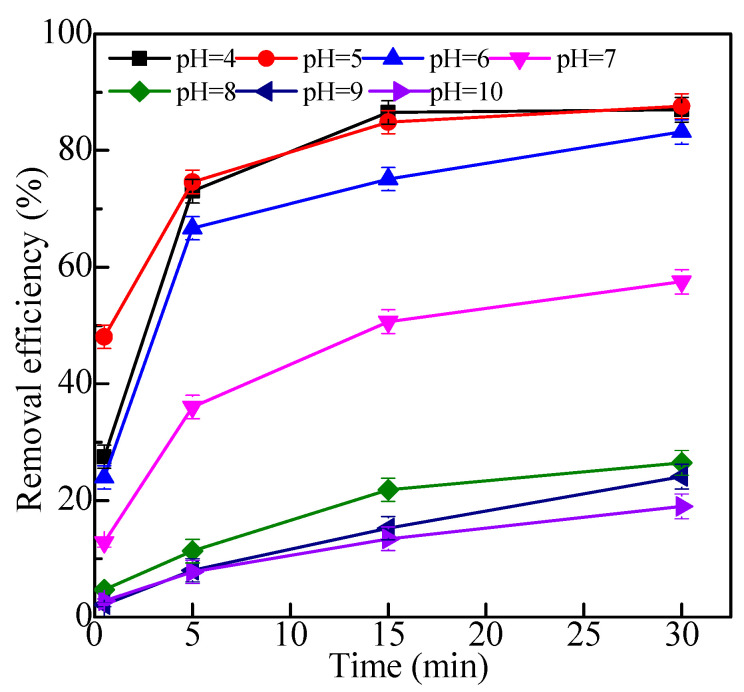
The effect of different pH on kaolin suspension treatment (the dosage was 2.5 mg/L, kaolin suspension concentration was 1.0 g/L, magnetic field intensity was 3800 G).

As shown in Figure 9, when the pH was 4–5, Fe_3_O_4_/HBPN showed a good removal performance of kaolin, and the removal effect was 88% in 30 min. On the contrary, Fe_3_O_4_/HBPN performed worse in alkaline conditions; the kaolin removal effect was less than 30% in 30 min. The results indicated that Fe_3_O_4_/HBPN had good flocculation characteristics in acidic environments. When the pH was 4–5, the zeta potential value of Fe_3_O_4_/HBPN was close to the negatively charged kaolin. At this time, the amino group (-NH_2_) was protonated into –NH_3_^+^, which made it easy to react with negatively charged kaolin, and it was integrated into larger magnetic flocs [72]. When the pH increased to alkaline, Fe_3_O_4_/HBPN still carried a positive charge, but the zeta potential value decreased significantly, and it was difficult for -NH_2_ to protonate. However, the zeta potential value of kaolin particles increased and carried a large amount of negative charge, which increased the electrostatic repulsion between particles and decreased the removal effect [73].

#### 2.2.3. Effects of Concentration of Kaolin Suspensions

**Figure 10:**
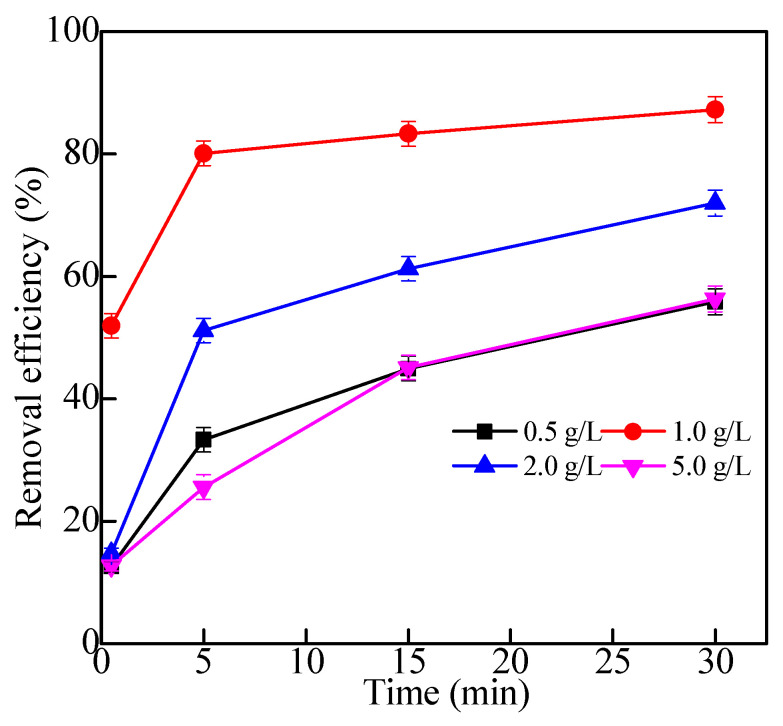
The treatment effect of different kaolin concentrations (the dosage was 2.5 mg/L, pH = 5.6, magnetic field intensity was 3800 G).

The removal effect of Fe_3_O_4_/HBPN at different kaolin concentrations is shown in Figure 10. When the kaolin concentration was 1.0 g/L, the removal performance of Fe_3_O_4_/HBPN was the best. The removal efficiency was 87% at 30 min, and it was significantly higher than other concentrations (0.5 g/L, 2.0 g/L, 5.0 g/L). This was because when the kaolin concentration was low, the Fe_3_O_4_/HBPN in solution was excessive, and the electrostatic repulsion among the flocs increased and inhibited the flocculation process. Moreover, the excessive kaolin concentration caused the amount of positive charge on the surface of Fe_3_O_4_/HBPN to be insufficient to neutralize the negative charge on the surface of kaolin particles.

#### 2.2.4. Effects of Magnetic Fields

**Figure 11:**
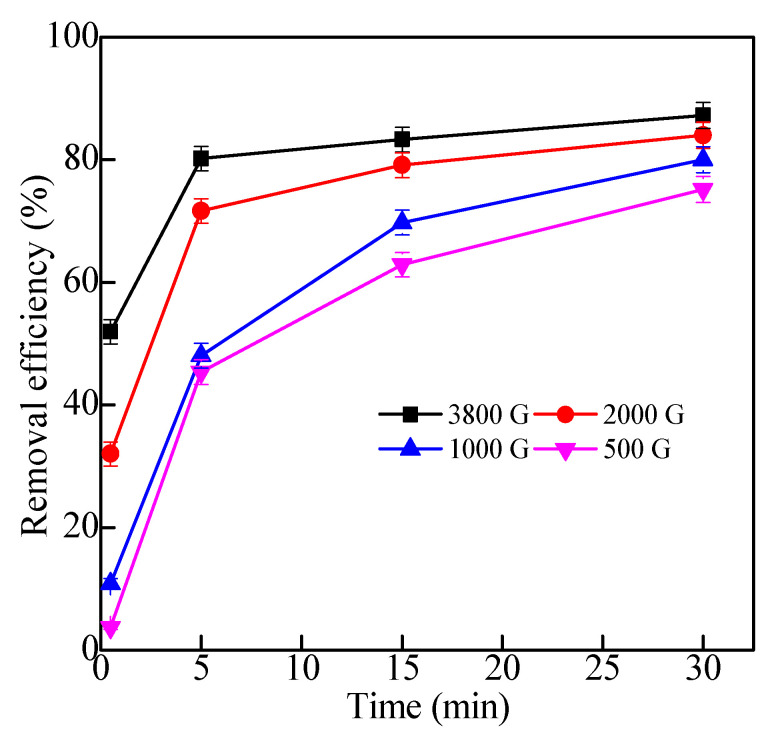
The effect of different magnetic fields intensity on kaolin suspension treatment (the dosage was 2.5 mg/L, pH = 5.6, kaolin suspension concentration was 1.0 g/L).

Figure 11 showed the removal effect of magnetic field strength. It was obviously observed that the removal effect of Fe_3_O_4_/HBPN increased significantly with the increase in magnetic field intensity. When the magnetic field intensity increased from 500 to 3800 G, the removal efficiency of kaolin went up to 52% at 0.5 min, while it rose to 80% at 5 min. For the magnetic particles, the addition of the magnetic field contributed to the rapid moving of magnetic flocs, colliding with each other during the process of agglomeration [74]. It created more opportunities for the subsequent bridging and charge neutralization. In the process of magnetic flocs movement, the magnetic force received was positively correlated with the magnetic field intensity and gradient, and the magnetic field force played a very important role in magnetic flocculation separation. Therefore, with the increase in magnetic field intensity, the removal rate gradually increases [75].

In general, the optimum reaction conditions were obtained by conducting the series experiments. These showed that the dosage of Fe_3_O_4_/HBPN was 2.5 mg/L, pH = 5.6, magnetic field intensity was 3800 G, and the kaolin suspension concentration was 1.0 g/L. By comparing with some other reported material applied on kaolin removal (Table 2), it was found that although the removal effect of Fe_3_O_4_/HBPN was slightly inferior to others, the dosage of Fe_3_O_4_/HBPN was much lower than other materials. That means Fe_3_O_4_/HBPN was more economical in the application process.

#### 2.2.5. The Actual Water Application

**Figure 12:**
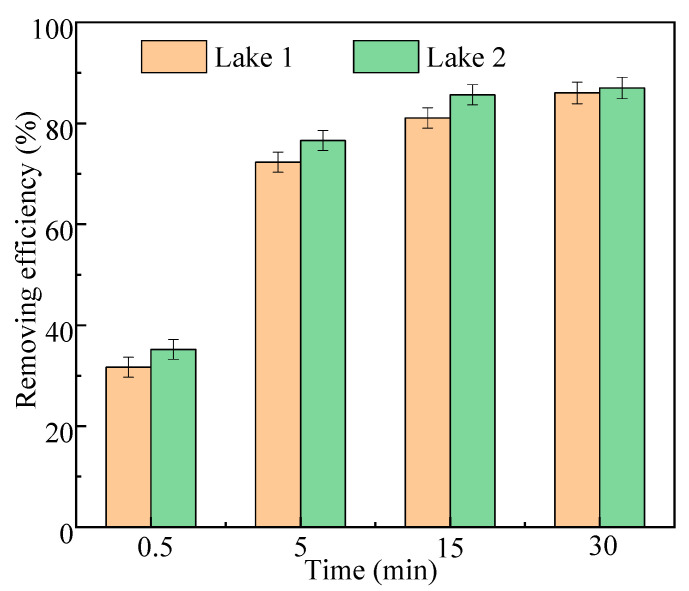
The actual water application effect in two lakes of Fe_3_O_4_/HBPN (the dosage was 2.5 mg/L, and the magnetic field intensity was 3800 G).

The water samples from two lakes were treated with Fe_3_O_4_/HBPN, respectively, and the results are shown in Figure 12. The removal efficiency for Lake 1 was 86% at 30 min, while it was 87% for Lake 2. It indicated that Fe_3_O_4_/HBPN still showed a significant flocculation effect for actual water due to the charge neutralization and adsorption bridging effects. The generated floc could quickly separate under the action of applied magnetic fields and obtain a high-turbidity removal rate. Moreover, there was no obvious difference regarding the treatment effect between the simulated and actual water (*p *> 0.05). When Fe_3_O_4_/HBPN was applied to actual water, the interference of complex water quality conditions was eliminated, and the flocculation effect was stable performance.

### 2.3. Recycling and Reusing

**Figure 13:**
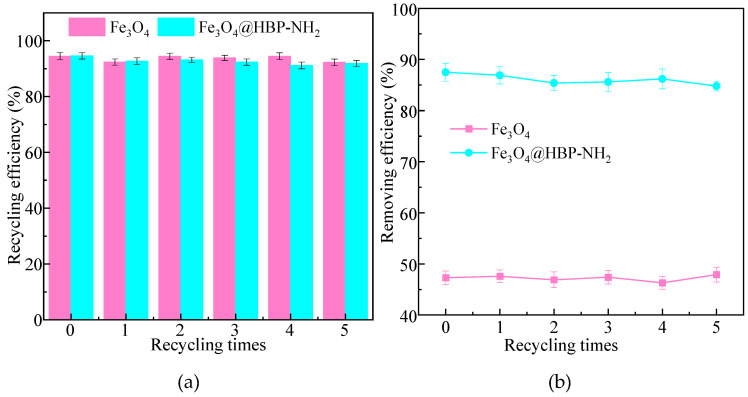
The recycling (**a**) and reusing (**b**) effect of Fe_3_O_4_ and Fe_3_O_4_/HBPN (pH = 5.6, kaolin suspension concentration was 1.0 g/L, magnetic field intensity was 3800 G).

The recovery of Fe_3_O_4_ particles from magnetic aggregates is an essential step, not only for particle recycle and reusing, but also for the downstream water treatment [77]. After Fe_3_O_4_/HBPN was used to treat kaolin suspensions, the material was separated from the solution via an external magnetic field. The recovered Fe_3_O_4_/HBPN was used again for the treatment of kaolin, and the operations were repeated five times to investigate the effect of regeneration and reusing. The recycling and reusing results are shown in Figure 13. Figure 13a shows the recycling efficiency of Fe_3_O_4_ and Fe_3_O_4_/HBPN. The recycling efficiency of Fe_3_O_4_ (92.3–94.5%) was slightly higher than that of Fe_3_O_4_/HBPN (91.2–93.2%), which is due to the fact that a small amount of functional groups loaded on the Fe_3_O_4_/HBPN surface fall off during separation. However, during the five recycling periods, there was no significant difference in RE regarding either Fe_3_O_4_ or Fe_3_O_4_/HBPN (*p* > 0.05). This showed that even if there was a small amount of loss in the application of Fe_3_O_4_/HBPN, the overall stability performed well.

The reusing performance of Fe_3_O_4_ and Fe_3_O_4_/HBPN is also shown in Figure 13b. As the control group, the removing efficiency of Fe_3_O_4_ was from 46.3% to 47.9%, and it showed no significant difference five times (*p* > 0.05). On the contrary, the removal efficiency of Fe_3_O_4_/HBPN before recycling was 87.5%, while the removal efficiency after 1–5 times recycling was in the range of 84.8%–86.9%. Although the removal efficiency before recycling was slightly higher than the subsequent recycled materials, there was no significant difference regarding the removal efficiency five times (*p* > 0.05). At the beginning of the application, a small amount of functional groups fell from the material surface and resulted in the slight variation after reusing. However, the stability of Fe_3_O_4_/HBPN remained after several times recycling and reusing, and the removal efficiency stayed at a relatively stable level.

## 3. Materials and Methods

### 3.1. Materials

Ferrous sulfate heptahydrate (FeSO_4_·7H_2_O), iron chloride hexahydrate (FeCl_3_·6H_2_O) and kaolin were purchased from Sinopharm Chemical Reagent Co., Ltd. (Shanghai, China). Ammonia (NH_3_·H_2_O), methanol, ethanol, methyl acrylate and diethylenetriamine were obtained from Shanghai Macklin Biochemical Technology Co., Ltd. 3-aminopropyl trimethoxysilane (APTMs) was bought from Shanghai Aladdin Biochemical Technology Co., Ltd. All the chemical reagents were analytical grade and were used for without further pretreatment.

### 3.2. Synthesis of magnetic Fe_3_O_4_ nanoparticles

The synthesis process of magnetic Fe_3_O_4_ nanoparticles was used by the method of co-precipitation [78]. First, 2.7 g of FeSO_4_·7H_2_O and 5.7 g of FeCl_3_·6H_2_O (molar ratio: 2:1) were dissolved in 100 mL of deionized water. Then, the pH of the mixture solutions was adjusted to 10.0 by NH_3_·H_2_O solution, which was added dropwise and stirred vigorously on a magnetic stirrer at 25 °C. Once the mixture solution turned to black, the black precipitate was separated from the solution using a permanent magnet. Furthermore, the precipitate was heated to the temperature of 80 °C for 30 min and washed 3 times using alternate solutions of distilled water and ethanol. Then, the magnetic Fe_3_O_4_ nanoparticles were obtained and free-dried.

### 3.3. Preparation of Magnetic Fe_3_O_4_/HBPN Composites

Firstly, the magnetic Fe_3_O_4_ nanoparticles were dispersed in the solution of methanol (200 mL) using an ultrasonic bath for 30 min. Subsequently, 11.6 mL of APTMs was dropped to the stirring solution at 25 °C for 4 h, and the mixture was separated after intensive stirring, which involved washing with methanol. Next, 100 mL of methanol and 21.6 mL of methyl acrylate were added into the above mixture and stirred continuously for 7 h. Then, the magnetic mixture was separated and washed with methanol again. At last, 18.12 mL of diethylenetriamine and 25 mL of methanol were added into the magnetic mixture in a conical flask; afterwards, it was reacted in an oil bath at 65°C for 1 h and 140 °C for 2 h, respectively, until the methanol evaporated. The final product in the flask consisted of hyperbranched magnetic composite Fe_3_O_4_/HBPN.

### 3.4. Characterization of Fe_3_O_4_/HBPN

The Fourier transform infrared (FTIR) spectra were monitored by employing a VERTEX 70 spectrometer (Bruker, Germany). The materials were grounded with KBr (1:100) and then compressed to form tables. The X-ray diffraction (XRD) patterns were collected with a Shimadzu XRD-7000 instrument at a scan rate of 0.02°·S^−1^ with a 2θ range of 20°–80° and Cu Κα radiation (λ = 0.1542 nm). XPS spectra were obtained via an Escalab 250 Xi spectrometer (Thermo Fisher Scientific Inc., Waltham, MA, USA) with a monochromated source of X-rays (Al Kα, 1486.6 photo energy) as the excitation source. SEM measurements were conducted on a TESCAN MIRA LMS microscope equipped with energy-dispersive X-ray spectrometry (Xplore 30, Oxford, UK). The thermal behavior analyses were conducted in an N_2_ atmosphere between room temperature and 800°C at a rate of 10 °C·min^−1^ using a Q50 thermogravimetric analyzer (TA Instruments- Waters LLC, New Castle, DE, USA). The magnetic properties of the samples were measured using a LakeShore 7404S vibrational sample magnetometer (Lake Shore Cryotronics, Inc., Westerville, OH, USA). The thermal behavior analyses were conducted in an N_2_ atmosphere between room temperature and 800°C at a rate of 10 °C·min^−1^ using a Q50 thermogravimetric analyzer (TA Instruments- Waters LLC, New Castle, DE, USA).

### 3.5. The Magnetic Separation Experiment

In order to investigate the performance of Fe_3_O_4_/HBPN, the kaolin-simulated high-turbidity wastewater and natural water were employed in the research. The kaolin suspension was prepared using kaolin suspended in a 2.0 L volumetric flask with a concentration of 2.0 g/L (1410 NTU). Except for the pH experiment, the pH of the kaolin suspensions was adjusted to 6.0.

The magnetic separation process was conducted in a jar test apparatus (ZR4-6, Zhongrun Water Industry Technology Development co., Ltd., China). The magnetic composite was first added to 400 mL kaolin suspensions (1.0 g/L) and then stirred for 1 min at 500 rpm; then, the beaker was placed inside a magnetic field created by a cubic NdFeB permanent magnet (50 mm L × 50 mm W × 25 mm H) with a magnetic induction intensity of 0.38 T. During magnetic separation, a 10 mL sample was collected from 3 cm below the solution surface at different time intervals (0.5, 5, 15, 30 min) to determine the concentration of kaolin. The separating efficiency (SE) was calculated using Equation (1):

(1) $$\mathrm{Separating}\mathrm{efficiency}(\%)=\frac{x_{0}-x_{t}}{x_{0}}$$

where *x_0_* (mg/L) and *x_t_* (mg/L) denote the initial kaolin concentration and kaolin concentration at time *t*, respectively.

The magnetic separation process was first tested at different dosages of magnetic composite Fe_3_O_4_/HBPN (1.0–20.0 mg/L). To investigate the effects of pH on separating, the pH of kaolin solution was adjusted in the range of 4–10, which using 1.0 mol/L HCl or 1.0 mol/L NaOH. Then, 0.5–5.0 g/L of kaolin concentrations was applied to explore the treatment effect. Different magnetic fields intensity of 500–3800 G were selected to test the kaolin removal effect. In the actual aqueous samples, the samples were taken freshly from two lakes in Luoyang City. To retain the accordant turbidity of the kaolin-simulated sample, moderate kaolin was added into the actual aqueous sample directly, keeping the turbidity at 1350–1450 NTU.

### 3.6. Recycle and Reuse of Fe_3_O_4_/HBPN

After the magnetic separation experiment, the supernatant was thoroughly removed from the beakers using a permanent magnet. The initial dosage of Fe_3_O_4_/HBPN was 2.5 mg/L. The magnetic aggregates were collected from all reaction vessels and dispersed in 5 mL of deionized water; then, the kaolin particles were detached from Fe_3_O_4_/HBPN composites by employing an ultrasonic generator (50 Hz, 1200 W) for 1 min. The recycled Fe_3_O_4_/HBPN composites were collected using the magnet (3800 G) and washed three times with deionized water. The recycled wet Fe_3_O_4_/HBPN composites were freeze-dried for further magnetic separation experiments. After weighing, Fe_3_O_4_/HBPN was evenly added to each reaction vessel to repeat the experiment. The recycling efficiency of Fe_3_O_4_/HBPN was calculated using Equation (2):

(2) $$\mathrm{recycling}\mathrm{efficiency}(\%)=\frac{m_{i,recovery}}{m_{i,dosing}}$$

where *m_i, recovery_* (mg) means the recovering weight of Fe_3_O_4_/HBPN, and *m_i, dosing_* (mg) means the dosing weight of Fe_3_O_4_/HBPN at dosing time *i* (*i* = 0, 1, 2…5).

### 3.7. Analytical Methods

Zeta potentials were measured in a model environment (distilled water, pH 2–12) at 25 °C using a Zetasizer Nano (2000HSA, Malvern, UK). Measurements were taken for kaolin (1.0 g/L), Fe_3_O_4_ (2.5 mg/L), Fe_3_O_4_/HBPN (2.5 mg/L) and recovered Fe_3_O_4_/HBPN. The supernatant after flocculation was detected directly. The pH of the solution was adjusted by 0.1 mol/L HCl and 0.1 mol/L NaOH, and the pH value was detected using a digital pH meter (PB-10, BSISL, China).

### 3.8. Statistical Analysis

The experiment data were analyzed using IBM SPSS 20 (SPSS Inc., Chicago, IL, USA). One-way analysis of variance (ANOVA) was employed to determine significant differences. A value of *p* < 0.05 was considered to be significantly different.

## 4. Conclusions

A kind of magnetic composites (Fe_3_O_4_/HBPN) was obtained by the embedded assembly of a three-dimensional amino-terminal hyperbranched polymer and Fe_3_O_4_. The –C–NH_2_ or –C–NH perssad of HBPN was linked with Fe_3_O_4_ particles by N–O bonds, which changed the physicochemical characteristics of naked Fe_3_O_4_. The shape and size of Fe_3_O_4_/HBPN showed a different and uneven distribution; the particles clumped together and were coated with an oil-like film. Meanwhile, Fe_3_O_4_/HBPN showed a positive charge in the pH range 4–10 and exhibited superparamagnetic properties. In the treatment of high-turbidity wastewater, Fe_3_O_4_/HBPN performed best on the kaolin suspension under the conditions of adding a dosage of 2.5 mg/L, pH = 4–5, the kaolin concentration of 1.0 g/L, and the magnetic field of 3800 G. Whether using simulated wastewater or actual water, the removal efficiency reached 86%. The recycle efficiency of Fe_3_O_4_/HBPN was in the range of 91.2%–93.2%, while the removal efficiency of kaolin suspension achieved 84.8% after five recycling and reuse cycles. These results show that Fe_3_O_4_/HBPN has strong structural stability for the efficient treatment of high-turbidity wastewater.

## Figures and Tables

**Table 1:** The EDS value of Fe_3_O_4_ and Fe_3_O_4_/HBPN.

Fe_3_O_4_				Fe_3_O_4_/HBPN									
O		Fe		C		N		O		Si		Fe	
Wt%	At%	Wt%	At%	Wt%	At%	Wt%	At%	Wt%	At%	Wt%	At%	Wt%	At%
23.90	52.30	76.10	47.70	13.75	28.17	5.93	10.42	23.03	35.44	1.64	1.44	55.65	24.53
23.62	51.91	76.38	48.09	20.86	39.18	6.93	11.17	19.57	27.60	1.95	1.57	50.69	20.48
29.93	59.85	70.07	40.15	15.40	28.88	6.77	10.88	28.03	39.45	1.76	1.41	48.05	19.38

**Table 2:** The comparison of reported materials treated with kaolin.

Materials	Dosage	Treatment Capacity	Conference
Fe_3_O_4_/SiO_2_	1.0 g/L	93.8%	[15]
CE-PEI	0.15 mg/mL	98.2%	[71]
CPAMF	0.24 g/L	92.4%	[13]
FS@CTS-P	150 mg/L	92.54%	[76]
Fe_3_O_4_/HBPN	2.5 mg/L	87%	This paper

## Data Availability

The data presented in this study are available on request from the corresponding author.

## Sample Availability

Not available.
